# Solanidine metabolic ratios for CYP2D6 phenotyping after a standardized potato meal

**DOI:** 10.3389/fphar.2026.1886427

**Published:** 2026-10-02

**Authors:** Julian Peter Müller, Daniel Friske, Mohammed Hasoumi, Bob Miller, Jens Andreas Sarömba, Jolanta Tupiec, Pascal Koczera, Thea Laurentius, Thomas Clavel, Julia C. Stingl, Katja S. Just

**Affiliations:** 1 Institute of Clinical Pharmacology, University Hospital RWTH Aachen, Aachen, North Rhine-Westphalia, Germany; 2 Functional Microbiome Research Group, Institute of Medical Microbiology, University Hospital RWTH Aachen, Aachen, North Rhine-Westphalia, Germany; 3 Department of Geriatric Medicine, University Hospital RWTH Aachen, Aachen, North Rhine-Westphalia, Germany; 4 Department of Geriatrics, Carl von Ossietzky University of Oldenburg, Oldenburg, Lower Saxony, Germany; 5 Department of Clinical Pharmacology and Pharmacoepidemiology, University Hospital Heidelberg, Heidelberg, Baden-Württemberg, Germany

**Keywords:** absorption, biomarker, CYP2D6, microbiome, phenotyping, solanidine

## Abstract

**Introduction:**

The recent identification of the diet-derived CYP2D6 biomarker solanidine, a potato constituent, enables the study of CYP2D6 activity *in vivo*. Although solanidine metabolic ratios (MR) were shown to be relatively stable over the day with high positive correlations to CYP2D6 phenotype and genotype, the influence of a solanidine-containing potato meal on solanidine metabolite plasma concentrations and MR is unclear.

**Methods:**

In this study with 20 healthy participants, not preselected for specific CYP2D6 genotypes, a standardized potato meal was administered after 3 days of potato-fasting (DRKS00031441). Blood sampling was conducted at 0, 0.5, 1, 1.5, 2, 3, 4, 5 and 29 h. Participants were genotyped for CYP2D6 and plasma concentrations of solanidine and its metabolites 4-OH-solanidine and 3,4-secosolanidine-3,4-dioic acid (SSDA) were measured by LC-MS/MS. MRs were formed by dividing metabolite by substrate concentrations at each sampling time point.

**Results:**

Solanidine and metabolite plasma concentrations showed no consistent increases. Solanidine plasma concentrations (29 h vs. baseline) increased in median 0.09 ng/mL (range: −0.37 to 1.43 ng/mL). Within the period of 29 h, the linear relationship of solanidine ln MRs to the CYP2D6 activity score (AS) improved over time after intake of the potato meal with the best association after 29 h (r^2^ = 0.56).

**Discussion:**

Changes in solanidine and metabolite plasma concentrations after the potato meal were variable, which could be attributed to interindividual absorption variability. CYP2D6 phenotyping may be improved by a previous potato meal at least 1 day before blood sampling. This may allow solanidine CYP2D6 phenotyping in populations with low dietary potato consumption.

## Introduction

CYP2D6 is one of the most important pharmacogenes and is involved in the metabolism of about 20%–30% of commonly administered drugs (Kamenski et al., 2020). CYP2D6 activity is highly variable due to genetic variants and non-genetic influences such as drug-drug interactions (DDIs). The recent identification of the dietary CYP2D6 biomarker solanidine is highly promising to assess the CYP2D6 phenotype *in vivo* (Magliocco et al., 2021; Tay-Sontheimer et al., 2014). Solanidine and its metabolites as CYP2D6 biomarkers allow to study CYP2D6 phenoconversion in patient cohorts (Sarömba et al., 2025), and may be used in the implementation of precision medicine by complementing CYP2D6 genotyping (Storset et al., 2025), or by detection of DDI effects on CYP2D6 activity.

Solanidine is a steroidal alkaloid that is taken up by consumption of potatoes and potato products (Harvey et al., 1985). In potatoes, solanidine is present predominantly in its glycosylated forms of α-solanine and α-chaconine (Hellenäs et al., 1992; Keuter et al., 2024), which together comprise roughly 95% of glycoalkaloids present in potatoes (Knuthsen et al., 2009). Studies in the pig cecum model suggest that deglycosylation of α-solanine and α-chaconine occurs by the gut microbiome (Kasimir et al., 2023). Absorption of the free steroidal alkaloid solanidine is increased compared to the glycoalkaloids as suggested by studies in Caco-2 cell monolayers (Keuter et al., 2024). After absorption, solanidine is specifically metabolized by CYP2D6 to 4-OH-solanidine and 3,4-secosolanidine-3,4-dioic acid (SSDA) among other metabolites (Magliocco et al., 2021; Behrle et al., 2022; Kiiski et al., 2024). Metabolic ratios (MR) of 4-OH-solanidine/solanidine and SSDA/solanidine were shown to correlate with the metabolism of CYP2D6 metabolized drugs such as dextromethorphan, risperidone, tamoxifen and metoprolol (Magliocco et al., 2021; Wollmann et al., 2023a; Medwid et al., 2024; Müller et al., 2024; Sarömba et al., 2026). In addition, the MR of 4-OH-solanidine/solanidine was able to reliably identify CYP2D6 poor metabolizers (PM) (Wollmann et al., 2023b).

In a previous study, we analyzed the stability of solanidine, its metabolites, and the corresponding MRs over a day and demonstrated a correlation of the MR SSDA/solanidine with the area under the curve (AUC) of MR α-hydroxymetoprolol/metoprolol at each of the measured time points even after exclusion of CYP2D6 PM subjects (Müller et al., 2024). However, in this study, like in other studies showing correlations of drug metabolism with solanidine MRs, the dietary intake of potato products was not assessed (Magliocco et al., 2021; Wollmann et al., 2023a; Medwid et al., 2024; Müller et al., 2024).

Although the effect of the dietary intake of potatoes and potato-products on solanidine serum levels has been described previously (Hellenäs et al., 1992), to this date there are no studies that specifically address the dietary intake of solanidine in relation to the CYP2D6 genotype and its influence on solanidine, its metabolites, and the resulting MRs over time. To address this knowledge gap, we performed a kinetic study with 20 subjects that received a solanidine-containing standardized potato meal to study effects on solanidine MRs and CYP2D6 phenotyping.

## Materials and methods

### Kinetic study

We conducted a kinetic study with 20 healthy volunteers, not preselected for specific CYP2D6 genotypes. In a pre-visit, volunteers were checked for eligibility to be enrolled in the study. When meeting the inclusion criteria, 7 mL blood in ethylenediaminetetraacetic acid (EDTA) containing tubes were drawn for CYP2D6 genotyping. Healthy volunteers eligible for enrollment were informed to refrain from consuming potato-containing products for 3 days prior to the start of the study. The study participants presented to the study fasted in the morning. On the study day, all participants received a meal of 5 potato fritters. Blood sampling was performed at 0, 0.5, 1, 1.5, 2, 3, 4, 5 and 29 h after the potato-containing meal using a peripheral venous catheter. To this end, 7 mL blood was drawn into EDTA containing tubes. To generate plasma, the blood samples were centrifuged for 10 min at 2,000 *g*. Plasma was subsequently aliquoted and was stored at −32 °C until analysis. Given the evidence that solanidine metabolism correlates well with CYP2D6 activity, no other CYP2D6 probe drug was used as a comparator in this study (Magliocco et al., 2021; Wollmann et al., 2023a; Medwid et al., 2024; Müller et al., 2024; Sarömba et al., 2026). A more detailed description of the study design including sample size calculation was published previously (Muller et al., 2026).

The study was approved by the ethical committee of the medical faculty of the RWTH Aachen University (approval number EK 23-087) and followed the Declaration of Helsinki. It was registered in the German Clinical Trials Register with the number: DRKS00031441.

### Food frequency questionnaire

The self-reported dietary intake of potatoes, potato products and other foods were assessed for 4 weeks prior to the first study day using a food frequency questionnaire (FFQ). The detailed results of the FFQ are given in Supplementary Table S1.

### Materials

Solanidine (CBS-FS76516, Biozol), 4-OH-solanidine (custom synthesized, for reference see (Sarömba et al., 2025)), dextromethorphan-d3 (D-071-1ML, Sigma-Aldrich), methanol (HPLC gradient grade, 8402.2500, J.T. Baker), water (LiChrosolv LC-MS grade, MC1153332500, Merck), formic acid (HiPersolv CHROMANORM LC-MS grade, 84.865.180, VWR), bovine serum (2203-010, Acila).

The following TaQman^TM^ assays were used for CYP2D6 genotyping (all ThermoFisher Scientific): *2 (rs16947, C__27102425_10), *3 (rs3574268, C__32407232_L0), *4 (rs3892097, C__27102431_D0), *6 (rs5030655, C__32407243_20), *8 (rs5030865, C_30634117C_K0), *9 (rs5030656, C__32407229_60), *10 (rs1065852, C__11484460_40), *14 (rs5030865, C_30634117D_M0), *17 (rs28371706, C___2222771_A0), *41 (rs28371725, C__34816116_20) and copy number variation assay (Hs00010001_cn) for duplication (xN) and deletion (*5).

### LC-MS/MS measurement of solanidine, 4-OH-solanidine and 3,4-secosolanidine-3,4-dioic acid

Plasma levels of solanidine, 4-OH-solanidine, and SSDA were determined using an UHPLC-MS/MS system consisting of an Agilent 1290 Infinity II UHPLC coupled to a Sciex QTRAP6500+ triple quadrupole mass spectrometer as previously described (Sarömba et al., 2025). Briefly, 50 µL plasma samples were protein-precipitated using 200 µL methanol with 0.1% formic acid (v/v) including the loading control dextrometorphan-d3 (1 ng/mL). Samples were vortexed for 10 s and centrifuged for 20 min at 17,000 *g* and 4 °C. 150 μL of the resulting supernatant were transferred into a new reaction tube and again centrifuged for 10 min at 17,000 *g* and 4 °C. Finally, 120 µL supernatant were transferred to an LC vial with insert. 14 μL of the supernatant were injected onto a Poroshell 120 EC-C18 column (1.9 µm, 2.1 × 50 mm, 699675-902, Agilent Technologies) with A: 0.1% formic acid (v/v) in water and B: methanol with a flow rate of 0.7 mL/min. The gradient program for liquid chromatography is shown in Supplementary Table S2. The analytes were detected using positive ion multiple reaction monitoring (MRM) with the following mass transitions (*m/z*): solanidine: 398.3 → 98.1 (quantifier), 398.3 → 382.3 (qualification), 4-OH-solanidine: 414.3 → 98.1 (quantifier), 414.3 →398.3 (qualification), 3,4-secosolanidine-3,4-dioic acid (SSDA): 444.3 → 370.3 (quantifier), 444.3 → 98.1 (qualification) and dextromethorphan-d3: 275.2 → 215.2 (loading control).

Calibration curves and quality control samples including solanidine and 4-OH-solanidine were constructed in charcoal stripped human plasma. Preparation of charcoal stripped human plasma was described previously (Sarömba et al., 2025). The calibration range for solanidine was from 0.01 to 10 ng/mL and for 4-OH-solanidine from 0.02 to 10 ng/mL with 8 and 7 non-zero calibrators, respectively. Calibration curves were weighted 1/x^2^ and the average accuracy (%) and coefficient of variation (CV in %) over all measured study batches was within ± 15% and lower than 15%, respectively. The average accuracy and precision data for all calibrator and quality control levels is given in Supplementary Table S3. Because no standard for SSDA was available, SSDA was quantified using the peak area as described previously (Sarömba et al., 2025; Müller et al., 2024).

### Determination of alkaloid content in the standardized potato meal

To determine steroidal alkaloid content in the potato meal, solanidine, and its glycosylated forms α-solanine and α-chaconine were determined by LC-MS/MS. The analytes were extracted with acidified acetonitrile and a simultaneous liquid-liquid extraction using anhydrous magnesium sulfate and sodium acetate as previously described with minor modifications (Liu et al., 2014). Modifications were: potato product samples were pestle by hand in liquid nitrogen and 1 mL of the upper phase were withdrawn, centrifuged for 20 min at 17,000 *g* and 4 °C of which 100 µL of the supernatant were transferred to a LC vial. 1 μL of the sample were injected for LC-MS/MS. The analytes were detected in positive ion MRM mode with *m/z* values of 868.4 → 398.5 *m/z* (quantifier) and 868.4 → 706.4 *m/z* (qualifier) for α-solanine and 852.4 → 706.5 *m/z* (quantifier) and 852.4 → 398.3 *m/z* (qualifier) for α-chaconine, respectively.

### CYP2D6 genotyping

The CYP2D6 genotype was assessed from whole blood samples using ThermoFisher Scientific TaQman^TM^ assays and a QuantStudio^TM^ 6 Pro qPCR-Machine (Applied Biosystems) as described previously (Sarömba et al., 2025). Genotyping was performed for the CYP2D6 variants *2, *3, *4, *6, *8, *9, *10, *14, *17 and *41. CYP2D6 copy number variation was assessed for duplication (xN) and deletion (*5). Clinical Pharmacogenetics Implementation Consortium (CPIC) guidelines were used to assign the CYP2D6 genotype-predicted phenotype and the activity score (AS) according to their diplotypes (Caudle et al., 2020).

### Post hoc analyses

The kinetic results of the study may suggest a potential absorption variability. Previous studies showed that solanidine is released from its glycosylated derivatives by the gut microbiome *in vivo* (Kasimir et al., 2023; Liou et al., 2024). Thus, two distinct human stool derived microbial communities were analyzed.

Stool sample 1 was collected and stored in GutAlive™ sampling buckets and delivered to the lab within 24 h. The stool material was homogenized and mixed 1:5 with anaerobic FMT media prior to storage at –80 °C until further use (Hitch et al., 2025). Stool sample 2 was generated by dissolving 50 g of freshly collected fecal material in 250 mL of 0.9% saline solution. After homogenization, the sample was centrifuged at 400 *g* for 5 min. The pellet was discarded, and supernatant again centrifuged at 6,000 *g* for 10 min. The resulting pellets were pooled in 22.5 mL 0.9% saline solution, 2.5 mL of 100% glycerol added and aliquots were prepared. To generate consistent microbiome-derived cryostocks as source material for experiments, the frozen stool samples were transferred anaerobically into Hungate tubes containing modified GAM broth (mGAM, Nissui Pharmaceutical Co., LTD.) within a Whatman Whitley A35 anaerobic workstation and incubated at 37 °C for 72 h to allow microbial communities to establish. Single-use cryostocks were then prepared from the expanded fecal slurries in Wilkins Chalgren Anaerobic medium (WCA) containing 20% glycerol and stored at – 80 °C. The use of human fecal samples was approved by the Ethics Committee of the University Hospital of RWTH Aachen, approval no. EK 23-055.

The cryostocks (described above) were thawed and incubated anaerobically in triplicate in Hungate tubes containing anaerobic mGAM medium for 48 h at 37 °C. Negative controls consisted of inactivated cultures generated by autoclaving an aliquot of cultivated communities at 121 °C for 20 min. To investigate the metabolism of α-chaconine or α-solanine, a final concentration of 1 µM of the respective compound was spiked into the culture medium. The reaction was terminated by adding 150 µL LC grade methanol to 50 µL sample. Samples were briefly vortexed and centrifuged for 20 min at 4 °C and 21,300 *g*. 50 μL of the resulting supernatant were diluted in 100 µL LC grade methanol and were transferred to a LC vial with insert. 5 μL of the sample were injected for LC-MS/MS analysis.

### Statistics and calculations

Solanidine and 4-OH-solanidine plasma concentrations were quantified using the respective analytical standards, SSDA plasma levels were determined using the peak areas. To generate MRs in the case of undetectable metabolite or solanidine levels, values were imputed by using half of the lower limit of quantification for solanidine (0.01 ng/mL) and 4-OH-solanidine (0.02 ng/mL) or the lowest measured values for SSDA as described previously (Magliocco et al., 2021; Sarömba et al., 2025; Müller et al., 2024). Only peaks with a signal/noise ratio above 10/1 were integrated for SSDA quantification.

The AUC_0–29h_ of solanidine, 4-OH-solanidine, and SSDA were calculated using Microsoft Excel 16 and the lin-lin method. Plasma concentrations were compared between baseline and t29. Standard deviations for plasma concentrations were calculated. Coefficients of variation (CV in %) were calculated for each participant from t0 to t29.

MRs were calculated by dividing metabolite by solanidine levels (metabolite/solanidine) and were ln-transformed. Time points at which neither solanidine, nor 4-OH-solanidine or SSDA were detected were excluded from MR calculations. Relationships of ln AUC_0–29h_ MR and ln MRs at single time points were tested against the genotype-predicted CYP2D6 activity score (AS) using linear regression analyses. Analyses were run with the whole cohort and after exclusion of PMs. P-values for single time points were corrected for multiple testing using the Bonferroni-Holm method. The self-reported potato consumption prior to the study was associated with the solanidine plasma concentrations.

Data were analyzed using Microsoft Excel 16 and GraphPad Prism 10. Linear regression analyses, Spearman correlation, Friedman test, Mann-Whitney U-test and Wilcoxon signed-rank test were performed using GraphPad Prism 10. P-values <0.05 were considered significant.

## Results

The study cohort consisted of 20 healthy subjects including all four CYP2D6 genotype-predicted phenotype groups. Detailed description of participants characteristics are shown in Supplementary Tables S4, S5.

Concentrations of α-solanine, α-chaconine, and solanidine per potato fritter were 11.17 mg/kg, 9.76 mg/kg, and 0.04 mg/kg, respectively. This resulted in a total alkaloid content of 20.97 mg/kg of wet weight potato fritter and corresponded to a dose of 6.29 mg alkaloids with the administration of the standardized potato meal of 5 potato fritters (in total 300 g) to each study participant.

The average plasma concentrations of solanidine before intake of the potato meal were 0.16 ± 0.39 ng/mL, which increased by 92% to an average concentration of 0.31 ± 0.53 ng/mL after 29 h. Likewise, plasma concentrations of 4-OH-solanidine increased by 57% from 0.79 ± 2.04 ng/mL to 1.24 ± 1.94 ng/mL. Average plasma levels of SSDA increased by 30% from 4.07*10^4^ ± 4.32*10^4^ to 5.30*10^4^ ± 5.07*10^4^ counts (Supplementary Figures S1A–C). Average plasma concentrations were the highest at t29 for all measured analytes (Supplementary Figures S1A–C). In addition, we assessed at which sampling point plasma concentrations of solanidine, 4-OH-solanidine, and SSDA were significantly different from baseline levels (t0). For solanidine, 4-OH-solanidine and SSDA, plasma concentrations were significantly different to baseline levels starting from 5, 29 and 4 h, respectively (Supplementary Figures S1A–C).

The individual plasma concentrations over time of solanidine, 4-OH-solanidine, SSDA, and the sum of solanidine and 4-OH-solanidine are shown in Figures 1A–D. Solanidine concentrations increased in median 0.09 ng/mL from t0 to t29, ranging from −0.37 ng/mL to 1.43 ng/mL (Figure 1A). 4-OH-solanidine levels showed a similar behavior like solanidine and increased in median for 0.20 ng/mL (range: −0.41 to 2.07 ng/ml) (Figure 1B). The median change in SSDA concentrations from t0 to 29 h was 8.2*10^3^ counts (range: −3.9*10^3^ to 7.2*10^4^ counts) (Figure 1C). The sum of the plasma concentrations of the quantified analytes solanidine and 4-OH-solanidine increased in median 0.39 ng/mL (range: −0.79 to 2.45 ng/mL) from t0 to t29 (Figure 1D). Interestingly, one CYP2D6 intermediate metabolizer (IM) participant showed elevated levels of solanidine, 4-OH-solanidine, and SSDA (Figures 1A–C). Notably, the participant’s solanidine plasma concentrations were even higher than those observed in the single poor metabolizer (PM) participant (Figure 1A).

**FIGURE 1:**
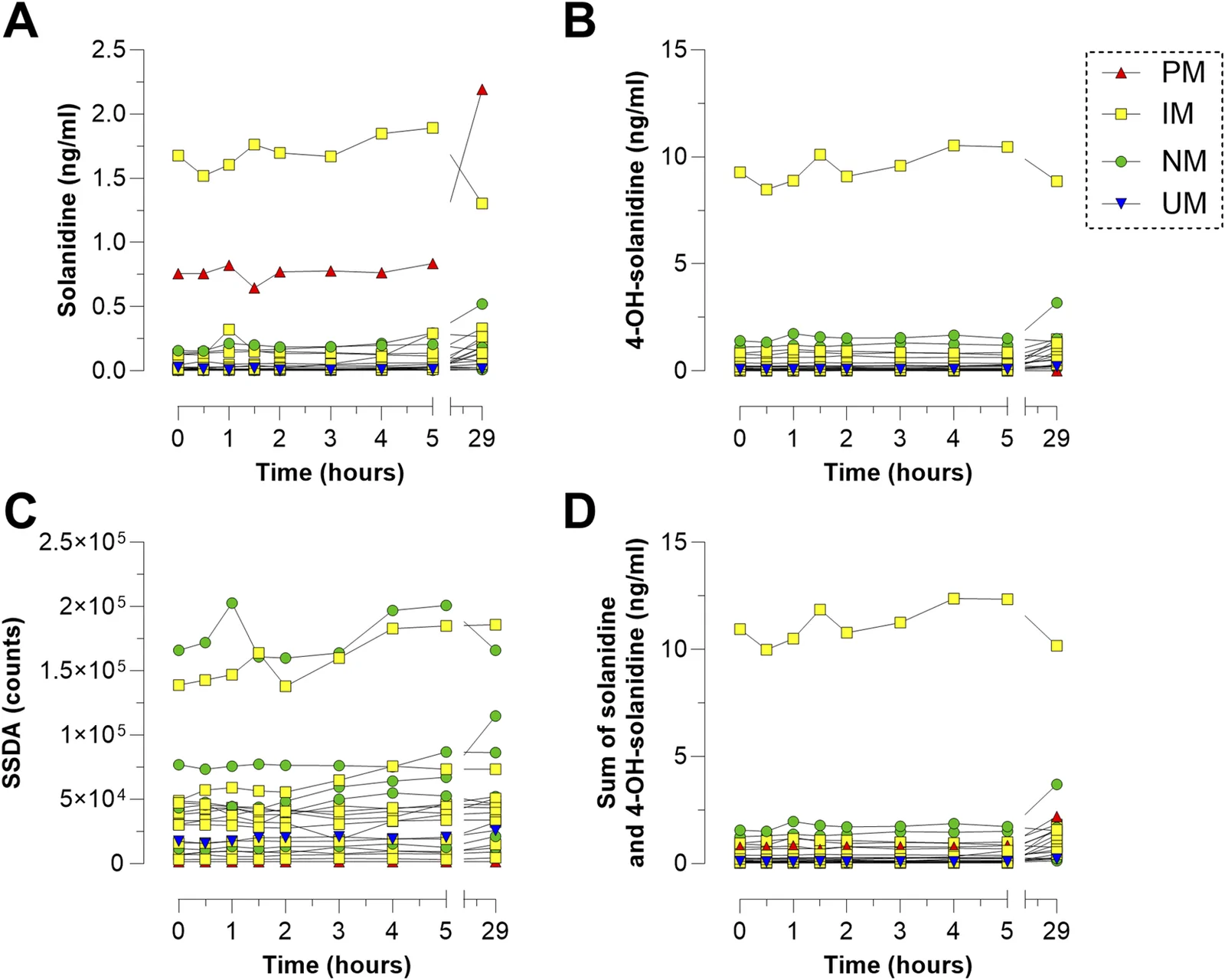
Measurements of solanidine, 4-OH-solanidine and SSDA in plasma samples from the study participants (n = 20) after the intake of a standardized potato glycoalkaloid containing meal. Individual plasma concentrations of **(A)** solanidine (ng/mL), **(B)** 4-OH-solanidine (ng/mL), **(C)** SSDA (counts) and **(D)** sum of solanidine and 4-OH-solanidine (ng/mL) are shown. Genotype-predicted CYP2D6 phenotypes are depicted by color coding. PM: poor metabolizer, IM: intermediate metabolizer, NM: normal metabolizer, UM: ultrarapid metabolizer, SSDA: 3,4-secosolanidine-3,4-dioic acid.

The individual CVs (in %) over time were used to quantify intraindividual variability and are presented as frequency distributions (Supplementary Figure S2). The CVs for solanidine and 4-OH-solanidine covered a broad range, reaching up to 249% and 266%, respectively. Variability of SSDA and the metabolic ratios was lower, with up to 75% CV, and their distributions were narrower (Supplementary Figure S2).

Solanidine MRs were able to differentiate between distinct CYP2D6 genotypes. The ln MR 4-OH-solanidine/solanidine and MR SSDA/solanidine showed a clear distinction between PM and non-PM participants over all measured time points, including the single IM with high solanidine plasma concentrations (Figures 2A,B). The linear relationship of the ln AUC_0–29h_ MR of 4-OH-solanidine/solanidine and ln AUC_0–29h_ MR of SSDA/solanidine was tested against the genotype-predicted CYP2D6 AS. Both associations were significant with r^2^ = 0.33, p-value = 0.008 and r^2^ = 0.53, p-value<0.001 for ln AUC_0–29h_ MR of 4-OH-solanidine/solanidine and ln AUC_0–29h_ MR of SSDA/solanidine, respectively (Figures 3A,B). The linear regression analysis remained significant after exclusion of the PM subject with r^2^ = 0.37, p-value = 0.006 and r^2^ = 0.45, p-value = 0.002 for ln AUC_0–29h_ MR of 4-OH-solanidine/solanidine and ln AUC_0–29h_ MR of SSDA/solanidine, respectively (Figures 3C,D). The same analysis was performed using the MR from single plasma sampling time points (Tables 1, 2). The ln MR SSDA/solanidine showed a significant linear relationship to the CYP2D6 AS at all measured time points after correction for multiple testing (Table 1). After exclusion of PM, the 29 h time point was significantly associated with CYP2D6 AS (Table 2). The performance of the ln MR 4-OH-solanidine/solanidine was less distinct and only the time point t29 showed a significant linear relationship with the CYP2D6 AS with and without exclusion of the PM subject (Tables 1, 2). Overall, the best associations with the CYP2D6 AS were observed 29 h after potato consumption with ln MR SSDA/solanidine (Tables 1, 2).

**FIGURE 2:**
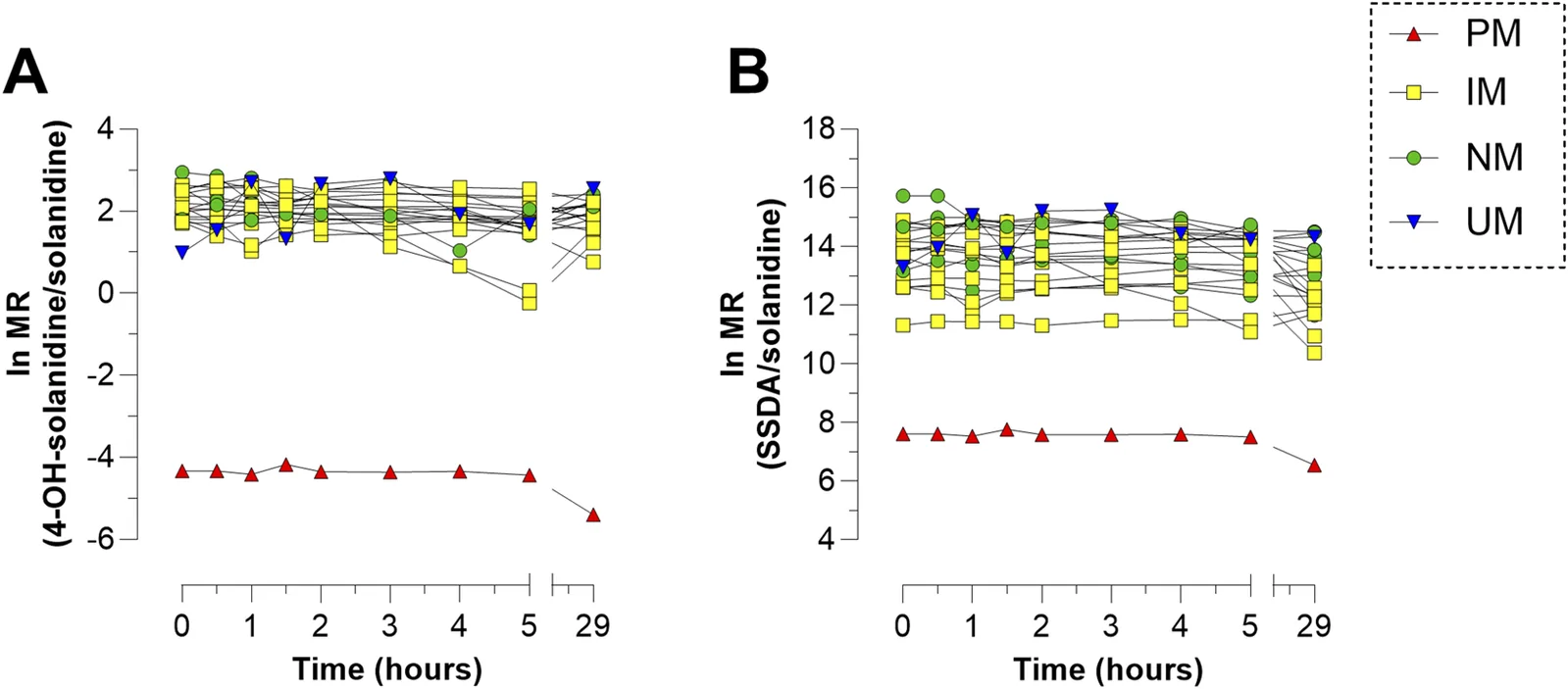
Time course of solanidine metabolic ratios (MR) from the individual study participants (n = 20) after intake of a standardized potato glycoalkaloid containing meal. **(A)** ln MR 4-OH-solanidine/solanidine and **(B)** ln MR SSDA/solanidine. Genotype-predicted CYP2D6 phenotypes are depicted by color coding. MR: metabolic ratio, SSDA: 3,4-secosolanidine-3,4-dioic acid, PM: poor metabolizer, IM: intermediate metabolizer, NM: normal metabolizer, UM: ultrarapid metabolizer

**FIGURE 3:**
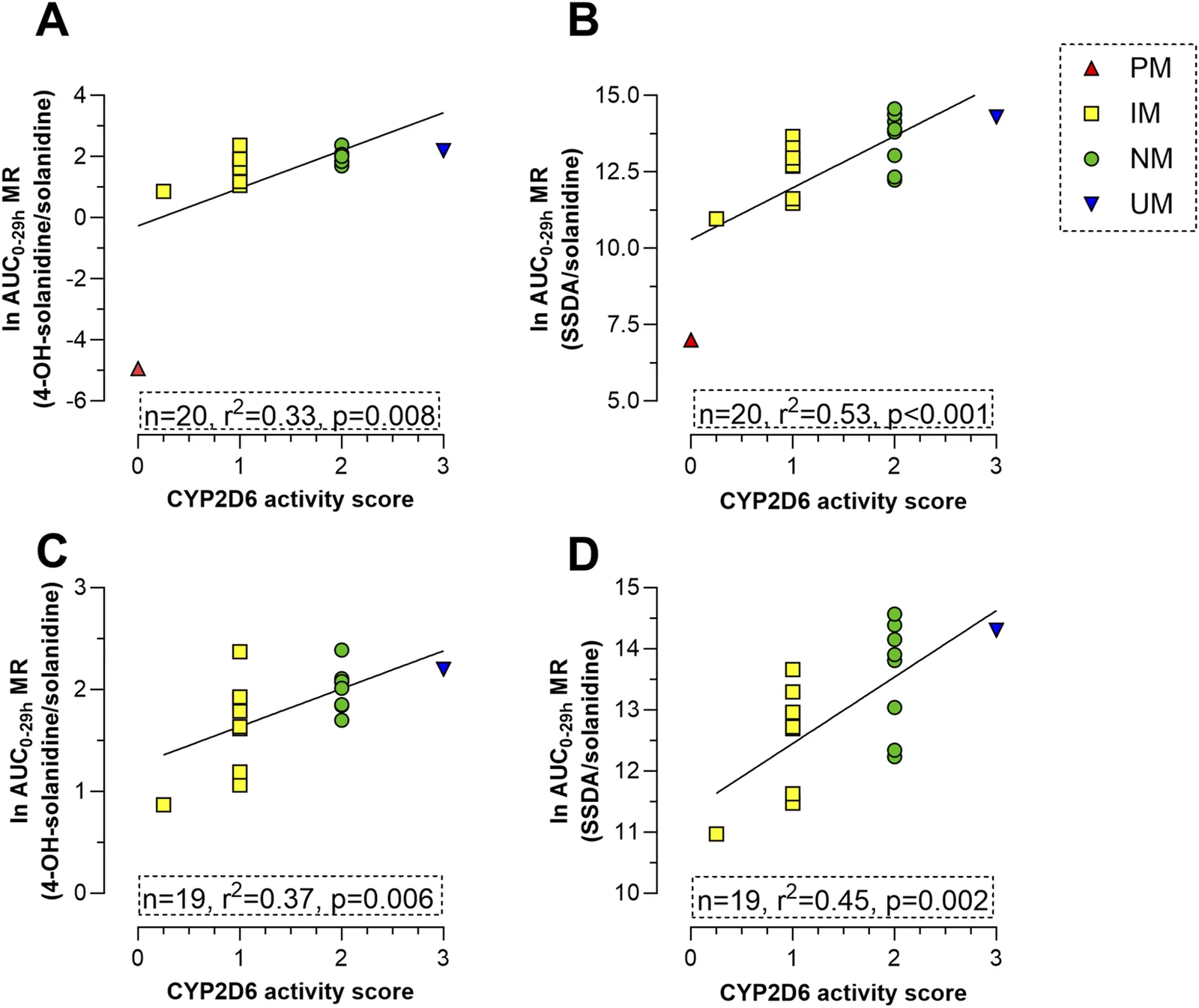
Linear regression analysis of solanidine area under the curve (AUC) metabolic ratios (MR) of the study participants with (n = 20) and without PM subject (n = 19) versus the genotype-predicted CYP2D6 activity score. **(A)** ln MR AUC_0–29h_ 4-OH-solanidine/solanidine, linear regression analysis: r^2^ = 0.33, p-value = 0.008. **(B)** ln MR AUC_0–29h_ SSDA/solanidine, linear regression analysis: r^2^ = 0.53, p-value<0.001. **(C)** ln MR AUC_0–29h_ 4-OH-solanidine/solanidine without PM subject, linear regression analysis: r^2^ = 0.37, p-value = 0.006. **(D)** ln MR AUC_0–29h_ SSDA/solanidine, linear regression analysis: r^2^ = 0.45, p-value<0.002. Genotype-predicted CYP2D6 phenotypes are depicted by color coding. AUC: area under the curve, MR: metabolic ratio, SSDA: 3,4-secosolanidine-3,4-dioic acid, PM: poor metabolizer, IM: intermediate metabolizer, NM: normal metabolizer, UM: ultrarapid metabolizer

**TABLE 1:** Linear regression analyses of ln-transformed solanidine metabolic ratios (MR) against the genotype-predicted CYP2D6 activity score of the study participants at each measured single plasma sampling time point.

Metabolic ratio (MR)	Time (hours)	0	0.5	1	1.5	2	3	4	5	29
ln MR 4-OH-solanidine/solanidine	r^2^	0.16	0.22	0.30	0.20	0.29	0.32	0.23	0.22	**0.37**
	Number of values	19	19	19	19	20	20	19	20	**20**
	Corrected p-value	0.1735	0.1735	0.1015	0.1735	0.1015	0.0776	0.1735	0.1735	**0.0405**
	Significance	ns	ns	ns	ns	ns	ns	ns	ns	*
ln MR SSDA/solanidine	r^2^	**0.27**	**0.33**	**0.38**	**0.30**	**0.39**	**0.43**	**0.36**	**0.36**	**0.56**
	Number of values	**20**	**20**	**20**	**20**	**20**	**20**	**20**	**20**	**20**
	Corrected p-value	**0.026**	**0.026**	**0.0234**	**0.026**	**0.0224**	**0.0128**	**0.026**	**0.026**	**0.001**
	Significance	*	*	*	*	*	*	*	*	***

P-values were corrected for multiple testing per condition (9 tests) using the Bonferroni-Holm method. Plasma sampling time points with a significant non-zero linear relationship after correction are depicted in bold. MR: metabolic ratio, SSDA: 3,4-secosolanidine-3, 4-dioic acid, PM: poor metabolizer, ns: not significant, * = p-value<0.05, ** = p-value<0.01, *** = p-value<0.001.

**TABLE 2:** Linear regression analyses of ln-transformed solanidine metabolic ratios (MR) against the genotype-predicted CYP2D6 activity score of the study participants at each measured single plasma sampling time point. The outlying CYP2D6 poor metabolizer (PM) subject was excluded from the analysis.

Metabolic ratio (MR)	Time (hours)	0	0.5	1	1.5	2	3	4	5	29
ln MR 4-OH-solanidine/solanidine (PM excluded)	r^2^	0.04	0.01**	0.15	0.00	0.21	0.24	0.02	0.03	**0.57**
	Number of values	18	18	18	18	19	19	18	19	**19**
	Corrected p-value	>0.999	>0.999	0.708	>0.999	0.3241	0.2568	>0.999	>0.999	**0.0018**
	Significance	ns	ns	ns	ns	ns	ns	ns	ns	**
ln MR SSDA/solanidine (PM excluded)	r^2^	0.08	0.15	0.22	0.12	0.24	0.31	0.19	0.19	**0.49**
	Number of values	19	19	19	19	19	19	19	19	**19**
	Corrected p-value	0.31	0.293	0.2616	0.31	0.2324	0.1096	0.293	0.293	**0.0072**
	Significance	ns	ns	ns	ns	ns	ns	ns	ns	**

P-values were corrected for multiple testing per condition (9 tests) using the Bonferroni-Holm method. Plasma sampling time points with a significant non-zero linear relationship after correction are depicted in bold. MR: metabolic ratio, SSDA: 3,4-secosolanidine-3, 4-dioic acid, PM: poor metabolizer, ns: not significant, * = p-value<0.05, ** = p-value<0.01, *** = p-value<0.001.

The self-reported potato consumption prior to the study was associated with the solanidine plasma concentrations. Baseline solanidine concentrations at t0 and the AUC_0–29h_ differed largely between participants with comparable weekly average potato consumption (Supplementary Figures S3A,B). The solanidine concentrations at t0 showed a weak but significant linear relationship with the self-reported weekly potato consumption measured using FFQs (r^2^ = 0.27, p-value = 0.018), while the solanidine AUC_0–29h_ did not (r^2^ = 0.19, p-value = 0.056). Of the 20 participants, 15 participants (75%) adhered to the instructions of the study protocol and did not consume potatoes within 3 days prior to the study. There was no statistically significant difference in solanidine plasma concentrations at t0 and the solanidine AUC_0–29h_ between participants that adhered to the study protocol and participants that admitted potato consumption (Supplementary Figures S3C,D).

The change in solanidine, 4-OH-solanidine, and SSDA concentrations from t0 to t29 was significant in the group (n = 15) that fasted potatoes within 3 days prior to the study (Supplementary Figure S4A). There were no significant differences of solanidine, 4-OH-solanidine, and SSDA plasma concentrations between t0 and t29 in the group (n = 5) that consumed potatoes directly prior to the study (Supplementary Figure S4B). The absolute increases (t0 to t29) in solanidine and 4-OH-solanidine plasma concentrations correlated significantly (ρ = 0.76, p-value<0.0001) (Supplementary Figure S5).

### Post hoc analyses

Solanidine was mainly present in the administered potato meal in the form of its glycosylated derivatives α-solanine and α-chaconine as measured by LC-MS/MS. We addressed whether deglycosylation of glycoalkaloids to solanidine may occur from human-stool derived microbial communities. In both analyzed samples, α-solanine and α-chaconine were fully converted to solanidine after 24 h. Heat-inactivation of the microbes fully abolished solanidine formation (Figure 4).

**FIGURE 4:**
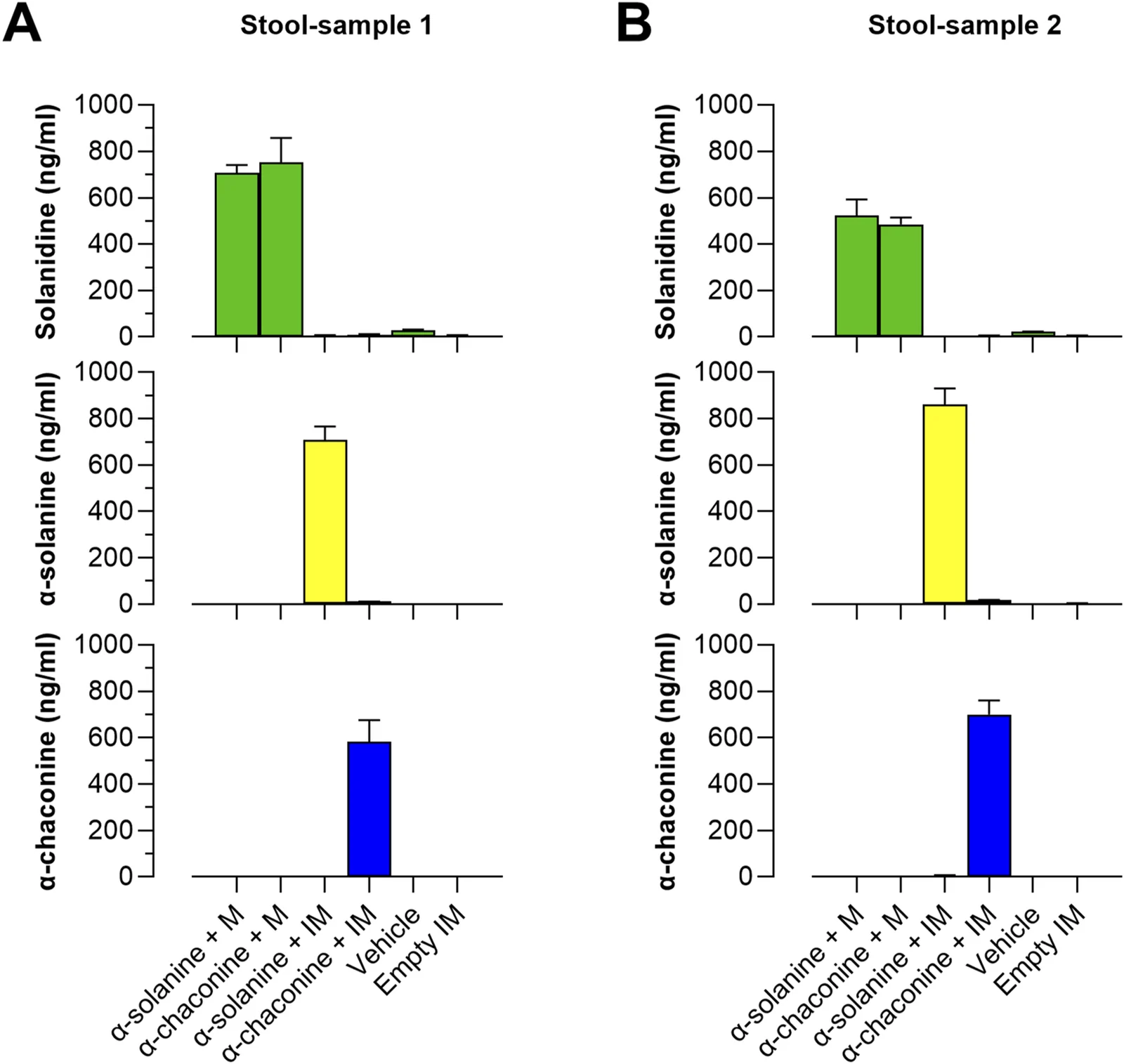
Release of solanidine from α-chaconine and α-solanine by human stool derived microbial communities. Microbial cultures derived from two individual patient stool samples 1 **(A)** and 2 **(B)** were incubated for 24 h at 37 °C with either 1 µM α-solanine or 1 µM α-chaconine. Solanidine, α-solanine and α-chaconine concentrations were measured in the samples using LC-MS. Data from a single experiment with three replicates per condition are shown for each stool sample. Data given as mean + SD. M: active microbial community, IM: inactivated microbial community.

## Discussion

In this study, plasma levels of solanidine and its metabolites showed a moderate postprandial increase after consumption of a standardized potato meal. Notably, the observed variability between individuals was only partly attributable to prior meals and CYP2D6 metabolism. Nevertheless, the identification of CYP2D6 PM was possible at all time-points irrespective of potato consumption. The association of solanidine MRs with the CYP2D6 genotype was best 29 h after administration of the standardized potato meal, suggesting that CYP2D6 phenotyping may be improved by a potato meal administered at least 1 day prior blood sampling.

In potato-derived products, solanidine is mainly present in its glycosylated forms, α-solanine and α-chaconine (Knuthsen et al., 2009). The total potato alkaloid content (α-solanine, α-chaconine and solanidine) in the administered potato meal was determined to be 20.97 mg/kg and corresponds well to previous reports of an alkaloid content of about 20–100 mg in potatoes (EFSA Panel on Contaminants in the Food Chain et al., 2020). This suggests that the amount of alkaloids administered by the used potato meal in this study may be similar to a typical potato-containing meal. The amount of potato alkaloids administered by the standardized potato meal was roughly 10-times lower (estimated amount: 0.09 mg/kg for a 70 kg person) than in previous studies assessing the uptake of α-solanine, α-chaconine and solanidine from the diet (Hellenäs et al., 1992). Hence, the glycoalkaloid amount administered in our study represents a more naturalistic scenario. While additional analysis of α-solanine and α-chaconine, would allow a deeper understanding of solanidine kinetics. As we focused on the analysis of solanidine MRs to describe CYP2D6 activity, we only measured these metabolites. We like to point out, that CYP2D6 PMs could be identified using solanidine MRs at any time point irrespective of solanidine kinetics measured in our study.

We observed only a slight increase in mean solanidine, 4-OH-solanidine, and SSDA plasma concentrations after the potato containing meal. The highest mean levels for all analytes were reached at the t29 sampling point. Changes in solanidine and metabolite plasma concentrations from t0 to t29 were highly variable with single participants even showing a decrease in solanidine and metabolite plasma concentrations over time. This observation was further supported by assessing the individual CVs (in %) over time. The CVs showed a wide distribution across participants, reaching values up to 249% (Supplementary Figure S2).

Solanidine plasma concentrations at t0 and the AUC_0–29h_ were not significantly different between participants that fasted on potatoes and those that admitted potato consumption prior to the study. This may be explained by a long elimination half-life of solanidine and the previous long-term diet of the participants (Harvey et al., 1985). Long-term dietary intake, as assessed by the FFQ, may also explain the high plasma concentrations of solanidine, 4-OH-solanidine, and SSDA observed in the single IM participant. This individual reported the second highest intake of potato products among all study participants with 1550 g/week. The absolute changes in plasma concentrations of solanidine, 4-OH-solanidine, and SSDA were statistically significant in the group that abstained from potato consumption according to the study protocol, but not in the group that consumed potato products immediately before the study. These results are difficult to attribute to prior dietary solanidine intake, as baseline solanidine levels were comparable between the two groups.

Absolute changes from t0 to t29 of solanidine and 4-OH-solanidine correlated well, suggesting that variable CYP2D6 activity is not the entire explanation for the observed variability in solanidine and metabolite plasma concentrations. Regardless of previous dietary intake and variable baseline levels, one would expect an overall increase in solanidine and metabolite plasma concentrations after the study meal, but in contrast some participants plasma levels decreased over time, even after summing solanidine and 4-OH-solanidine levels. This may suggest a variability in dietary solanidine absorption, which may be either due to variable amounts absorbed from the diet or a variable T_max_, e.g., due to delayed uptake.

The larger molecular size and higher polarity of the glycoalkaloids α-solanine and α-chaconine compared to solanidine, may lead to a lower uptake across the gut wall (Keuter et al., 2024). Previous studies suggest that glycoalkaloids are deglycosylated by the gut microbiome to form free solanidine (Kasimir et al., 2023). Solanidine exhibits greater permeability across the intestinal epithelium and is more readily absorbed than its glycosylated precursors (Keuter et al., 2024). Variable and interindividual differences in human gut microbiome composition could lead to individual differences in solanidine absorption from the diet. In our own complementary *in vitro* experiments we confirmed that solanidine is formed from α-chaconine and α-solanine by microbial cultures derived from human stool samples (Kasimir et al., 2023). The metabolism of α-chaconine and α-solanine to solanidine by the gut microbiome could be a potential factor for an individual absorption variability. In fact, incubation of α-solanine and α-chaconine with individual microbiome cultures derived from 30 human stool samples showed pronounced variability in deglycosylation activity and solanidine release (Liou et al., 2024). Next to a diet low in potatoes, individuals with lower deglycosylation activity within their gut microbiome may present with low or no detectable levels of solanidine due to decreased intestinal absorption. This however, needs further confirmatory studies. Our study points to an importance of regular potato-product consumption to enable robust CYP2D6 phenotyping using solanidine, and may offer an explanation for undetectable levels in some participants seen in larger study cohorts (Kiiski et al., 2024; Wollmann et al., 2023b). Importantly, because solanidine concentrations are used for normalization, variability in solanidine absorption is unlikely to influence the MRs used to phenotype CYP2D6. However, absorption variability may still be an important consideration when solanidine is administered via a potato meal. This may be particularly important in regions with lower habitual consumption of potato products, such as East Asia (Bae et al., 2026).

Our analyses showed that the correlation between solanidine MRs and the genotype-derived CYP2D6 activity score (AS) improved with increasing time after potato meal consumption. As expected from a dietary biomarker, regular intake of solanidine may increase its predictive performance to phenotype CYP2D6 activity. Simultaneously, this suggests that the administration of a single potato meal to individuals from populations with very low or no intake of dietary solanidine could be attempted to facilitate CYP2D6 phenotyping on the following day. This may avoid the need of CYP2D6 probe drug administration, which may lead to adverse drug reactions and/or toxicity (Magliocco et al., 2019; Jackson et al., 2023).

This study has limitations. We did not use an exogenous probe drug for CYP2D6 as a reference phenotype for the solanidine MRs, which may have shown stronger associations compared to the CYP2D6 AS, as previously shown (Magliocco et al., 2021; Wollmann et al., 2023a; Müller et al., 2024). Still, a significant linear relationship to the CYP2D6 AS derived from the genotype was observed and in line with previous reports, the ln MR SSDA/solanidine was more accurate in predicting the CYP2D6 AS than the ln MR 4-OH-solanidine/solanidine (Magliocco et al., 2021; Müller et al., 2024). Due to an intertwined study design a large plasma sampling gap between 5 and 29 h existed (Muller et al., 2026), which hindered to accurately detect potential C_max_ and t_max_ during this time frame. Although patients were asked to abstain from potato consumption, an influence of previous potato meals on changes in solanidine, 4-OH-solanidine, and SSDA could be even likely due to expected long plasma half-lives (Harvey et al., 1985). Hence, C_max_ and t_max_ may lay after 5 h. One old study focusing on potato toxicity measured an estimated t_max_ for solanidine between 8 and 25 h after potato ingestion (Hellenäs et al., 1992). An optimization of the sampling window would include blood sampling between this range. Unfortunately in our study, no stool samples were collected from the participants during the study, which may have allowed a direct comparison of individual microbiome composition and solanidine absorption. Further large-scale studies are required to assess the generalizability of the present findings. In addition, the use of an optimized sampling window would allow a more robust determination of the C_max_ and t_max_ of solanidine and its metabolites. Nevertheless, the current results provide a valuable and needed basis for future research in this area.

In conclusion our study shows interindividual variability of solanidine and metabolite plasma concentrations after a standardized potato meal. This variability cannot be fully explained by additional dietary intake prior to the study or by differences in CYP2D6 activity. Instead, this may suggest an absorption variability from the diet, e.g., by variable microbial release of solanidine from glycoalkaloids. Nevertheless, the use of MR adequately corrects for differences in solanidine exposure. By using the ln MR SSDA/solanidine, we demonstrate a significant linear relationship to the genotype-derived CYP2D6 activity at each plasma sampling time point. The correlation was best 29 h after the potato meal, suggesting that in populations with low exposure to solanidine, a single solanidine containing meal a day before the blood sampling may be sufficient to facilitate CYP2D6 phenotyping.

## Data Availability

The data presented in the study are deposited in a repository accessible under the following link: https://github.com/JulianPeterMueller/Solanidine-standardized-potato-meal.
