# Supplementary material for: Solanidine metabolic ratios for CYP2D6 phenotyping after a standardized potato meal
Source: Front Pharmacol. 2026 Oct 2;17:1886427. doi: 10.3389/fphar.2026.1886427 (PMC13671953; doi:10.3389/fphar.2026.1886427)
Supplement: Supplementary file 1 [file DataSheet1.pdf]

Table S1: Results from the food frequency questionnaire (FFQ). Study participants self-reported their average dietary intake in g/week.

Participant-No.	Potato products (g/week)	alcohol consumption (ml/week)	chinin containing drinks (ml/week)	grapefruit juice (ml/week)	citrus fruits (g/week)	other night shades (g/week)	special comments on nutrition/supplements
1	900	0	0	0	1680	1700	vegetarian
2	350	500	0	0	0	1000	vitamin D3 and B12, omega 3, creatine, protein powder
3	350	0	0	0	360	1385	protein products
4	350	500	0	0	240	930	protein products
5	265	200	0	0	120	990	vitamin D3, omega 3, FS, iron, iodide, protein powder
6	90	500	0	0	70	975	0
7	1550	2075	0	0	120*	1155	0
8	125	0	0	0	0	500	protein, creatine, carnitine, B-complex, magnesium
9	700	0	0	0	120	925	vegetarian
10	275	250	0	0	0	460	vitamin K2 and D3
11	825	1000	0	0	50	1025	whey protein
12	560	0	0	0	30	1100	vitamin C, vitamin D, vitamin B12, iron
13	700	0	0	0	0	2100	magnesium
14	490	0	0	0	0	1250	protein products
15	215	500	0	0	120	700	whey protein
16	220	0	0	75	0	100	multi vitamin, protein powder, creatine
17	750	0	0	0	120	200	protein powder, vitamin D
18	1830	0	0	0	300	1400	protein powder
19	1000	0	0	1000	200	1500	0
20	200	0	0	0	0	0	protein powder

If no information in g/week was given, the food stuffs were rated as follows: potato (per piece): 120 g. Citrus fruits: orange (per piece): 120 g, lemon (per piece): 120 g, lime (per piece): 70 g. Other night shades: tomatoes (as part of a dish): 250 g, tomatoes (per piece): 150 g, bell pepper (as part of a dish/per piece): 200 g, chili (per piece): 5 g, eggplant (as part of a dish): 175 g, eggplant (per piece): 350 g, * 600 ml orange juice

Table S2: Liquid chromatography gradient program for the measurement of solanidine, 4-OH-solanidine and 3,4-secosolanidine-3,4-diocic acid

Time (min)	%B
0	5
0.10	5
0.15	37
0.90	37
1.40	53
1.65	53
1.85	59.3
1.95	70
2.1	70
2.4	99
3.9	99
4.0	96.5
4.65	96.5
4.7	5
5	5

Table S3: Average accuracy (%) and coefficient of variation (CV in %) for solanidine and 4-OH-solanidine calibration and quality control samples over all measured study batches.

		Nominal concentration (ng/ml)	CV (%)	Av. Accuracy (%)
Solanidine	Calibrators	0.01	9.66	99.01
		0.02	5.57	107.35
		0.03	6.62	104.00
		0.06	6.70	86.10
		0.2	4.02	100.91
		1	4.50	108.55
		5	3.73	103.14
		10	5.24	94.60
	QC	0.01	15.93	112.46
		0.02	6.68	105.03
		0.04	5.31	91.60
		4.5	6.62	86.63
		9	5.51	94.97
4-OH-solanidine	Calibrators	0.02	4.31	103.53
		0.03	6.92	101.16
		0.06	6.59	86.42
		0.2	3.51	100.54
		1	3.95	108.34
		5	2.48	103.73
		10	2.59	96.29
	QC	0.02	5.14	103.29
		0.04	8.94	95.21
		4.5	4.84	88.92
		9	2.38	97.57

QC: quality control, CV: coefficient of variation

Table S4: CYP2D6 genotyping results with annotation of the CYP2D6 genotype-predicted phenotype and activity score.

Genotype-predicted phenotype	Subjects, n (%)	CYP2D6 diplotype (n)	CYP2D6 activity score (n)
PM	1 (5)	*4/*4x2 (1)	0 (1)
IM	10 (50)	*4/*41 (1)	0.25 (1)
		*1/*4 (5), *1/*3 (1), *2/*4 (3)	1 (9)
NM	8 (40)	*1/*1 (4), *1/*2 (3), *2/*2 (1)	2 (8)
UM	1 (5)	*1/*2x2 (1)	3 (1)

PM: poor metabolizer, IM: intermediate metabolizer, NM: normal metabolizer, UM: ultrarapid metabolizer, n: number

Table S5: Baseline data of the study participants.

Parameter	Subjects, n (%)	median (range)
female	6 (30)	
male	14 (70)	
adherence to study protocol	15 (75)	
age (years)		24 (22 - 27)
eGFR (ml/min/1.73m^2)		115.55 (97.6 - 130.2)
BMI (kg/m^2)		23.5 (19.7 - 28.1)

eGFR: estimated glomerular filtration rate, BMI: body-mass index

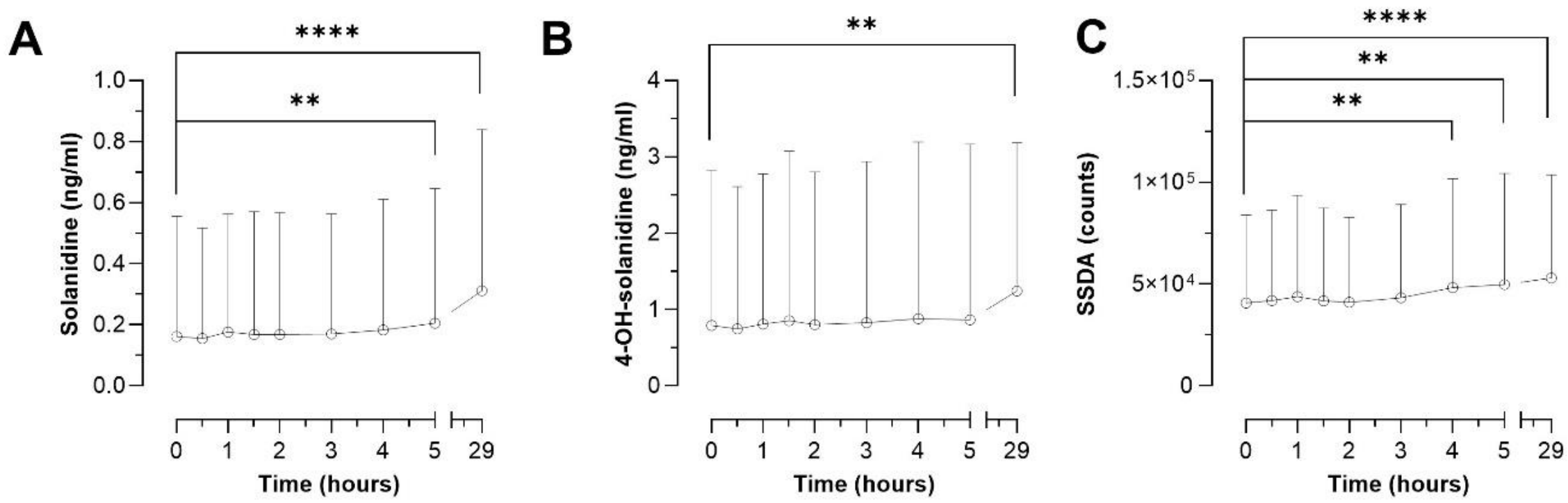

Figure S1: Mean solanidine, 4-OH-solanidine and SSDA plasma concentrations of the 20 study participants after intake of a solanidine and glycoalkaloids containing standardized potato-meal. A: Mean plasma concentrations of solanidine given in ng/ml. B: Mean plasma concentrations of 4-OH-solanidine given in ng/ml. C: Mean plasma concentrations of SSDA given in counts (arbitrary unit). Data given as mean + standard deviation. Significant deviation of solanidine and metabolite plasma concentrations from baseline levels (t0) were assessed using nonparametric Friedman test with Dunn´s post hoc test. Time points with significant difference to t0 in the multiple comparisons test are depicted by asterisks. *=p-value<0.05, **=p-value<0.01, ***=p-value<0.001, ****=p-value<0.0001. SSDA: 3,4-secosolanidine-3,4-dioic acid

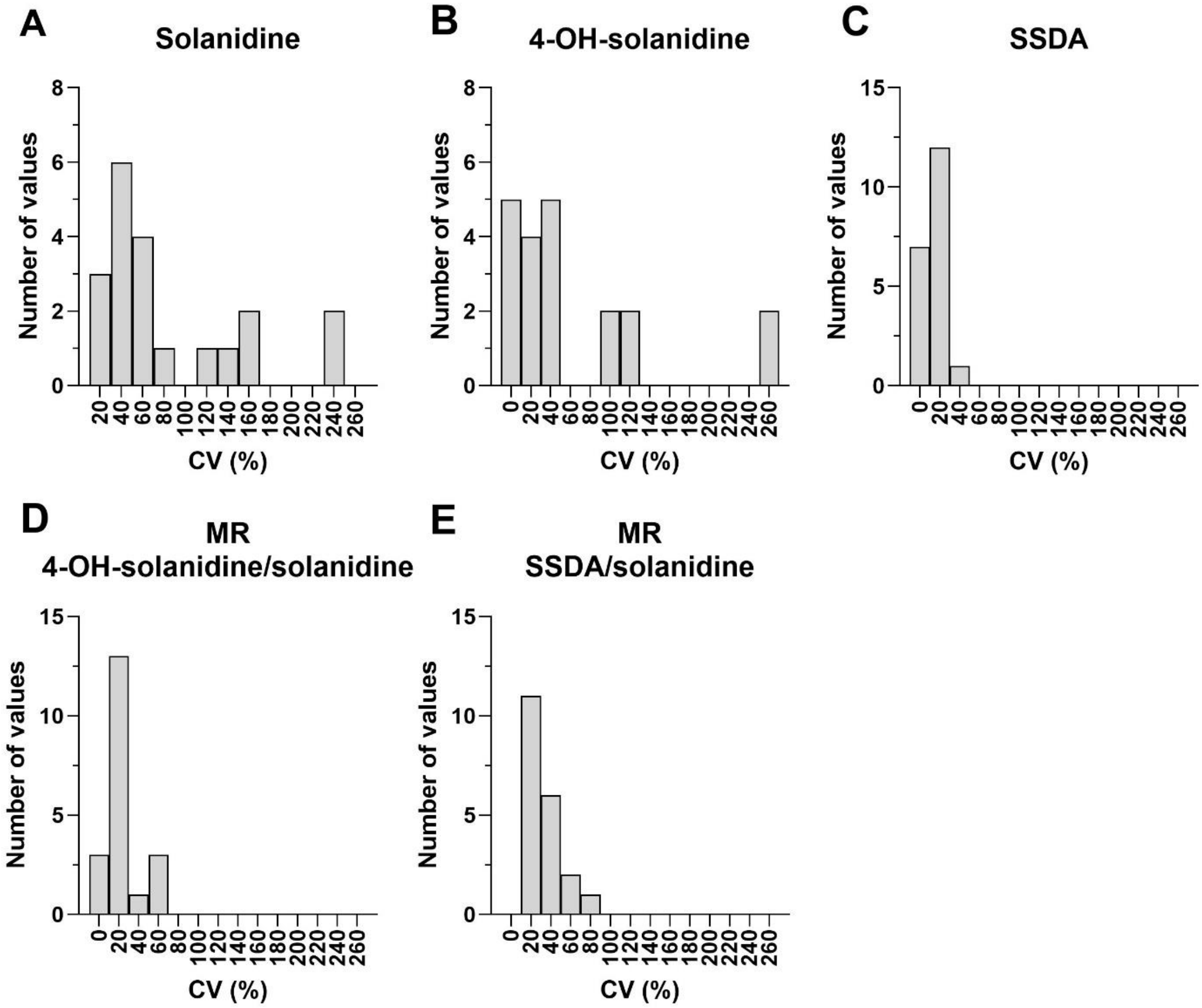

Figure S2: Frequency distribution of the individual coefficients of variation (CV in %) across all time points from t0 to t29 (n=9 values) for A: solanidine, B: 4-OH-solanidine, C: SSDA, D: MR 4-OH-solanidine/solanidine and E: MR SSDA/solanidine. Number of values and the bin centers are given. CV (%): coefficient of variation given in %, SSDA: 3,4-secosolanidine-3,4-dioic acid, MR: metabolic ratio

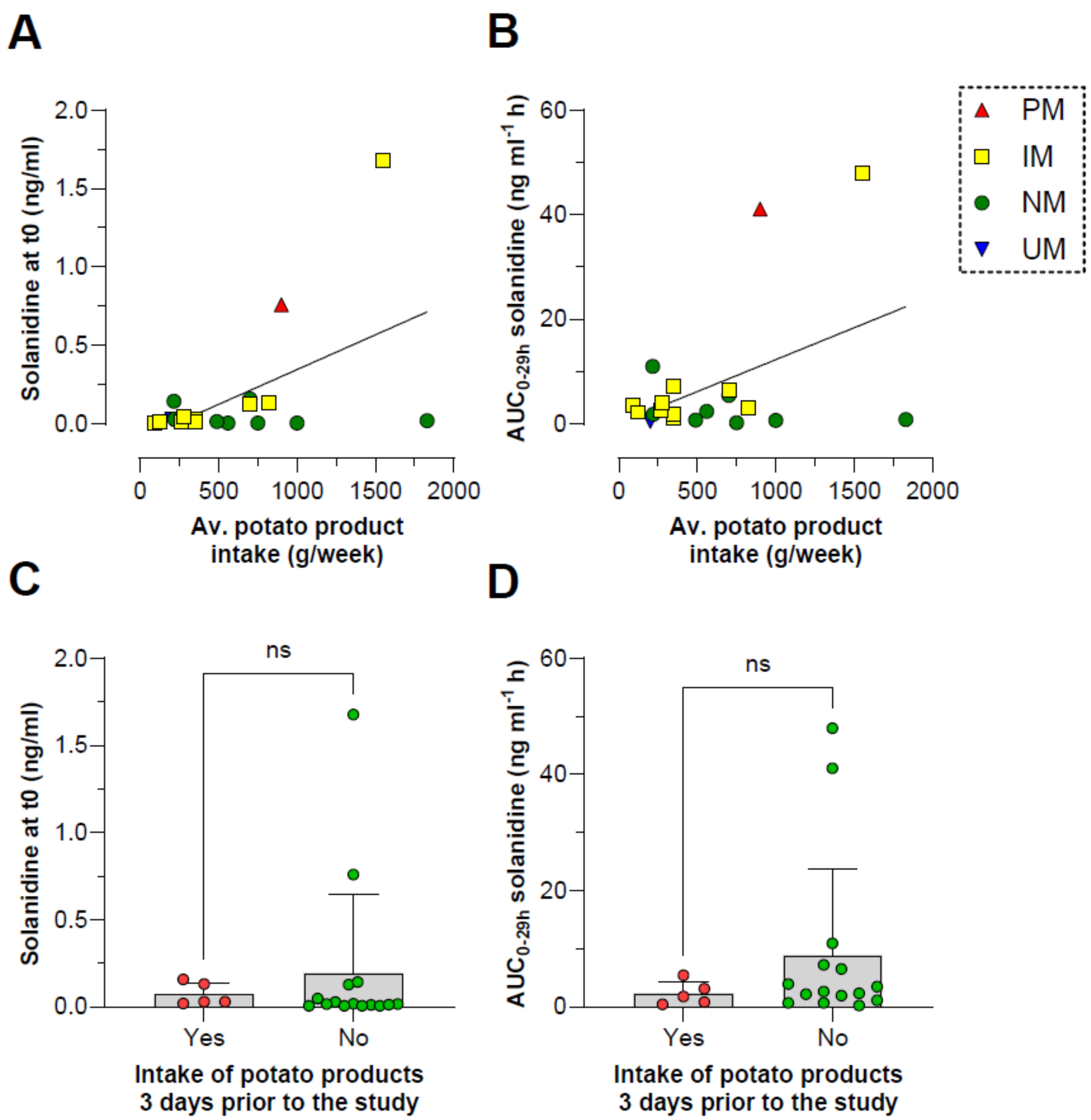

Figure S3: Solanidine plasma concentrations in relation to the self-reported diet of the study participants. A: Solanidine concentrations (ng/ml) measured in plasma of the participants (n=20) at t0 prior to administration of the potato-meal compared to the average potato intake (g/week) over the last four weeks according to the food frequency questionnaire (FFQ). Linear regression: r^2 = 0.27 and a p-value of 0.018. B: Solanidine $AUC_{0\text{-}29h}$ (n=20) in relation to the average potato product intake (g/week) over the last four weeks according to the food frequency questionnaire (FFQ). Linear regression: r^2 = 0.19 and a p-value of 0.056. The individual CYP2D6

genotype-predicted phenotypes are assigned. C: Solanidine concentrations (ng/ml) measured in plasma at t0 and D: Solanidine $AUC_{0\text{-}29h}$ compared to self-reported potato product consumption in the last three days prior to the study. Yes: n=5, No: n=15. The difference between groups was tested using Mann-Whitney test. ns: not significant, PM: poor metabolizer, IM: intermediate metabolizer, NM: normal metabolizer, UM: ultrarapid metabolizer

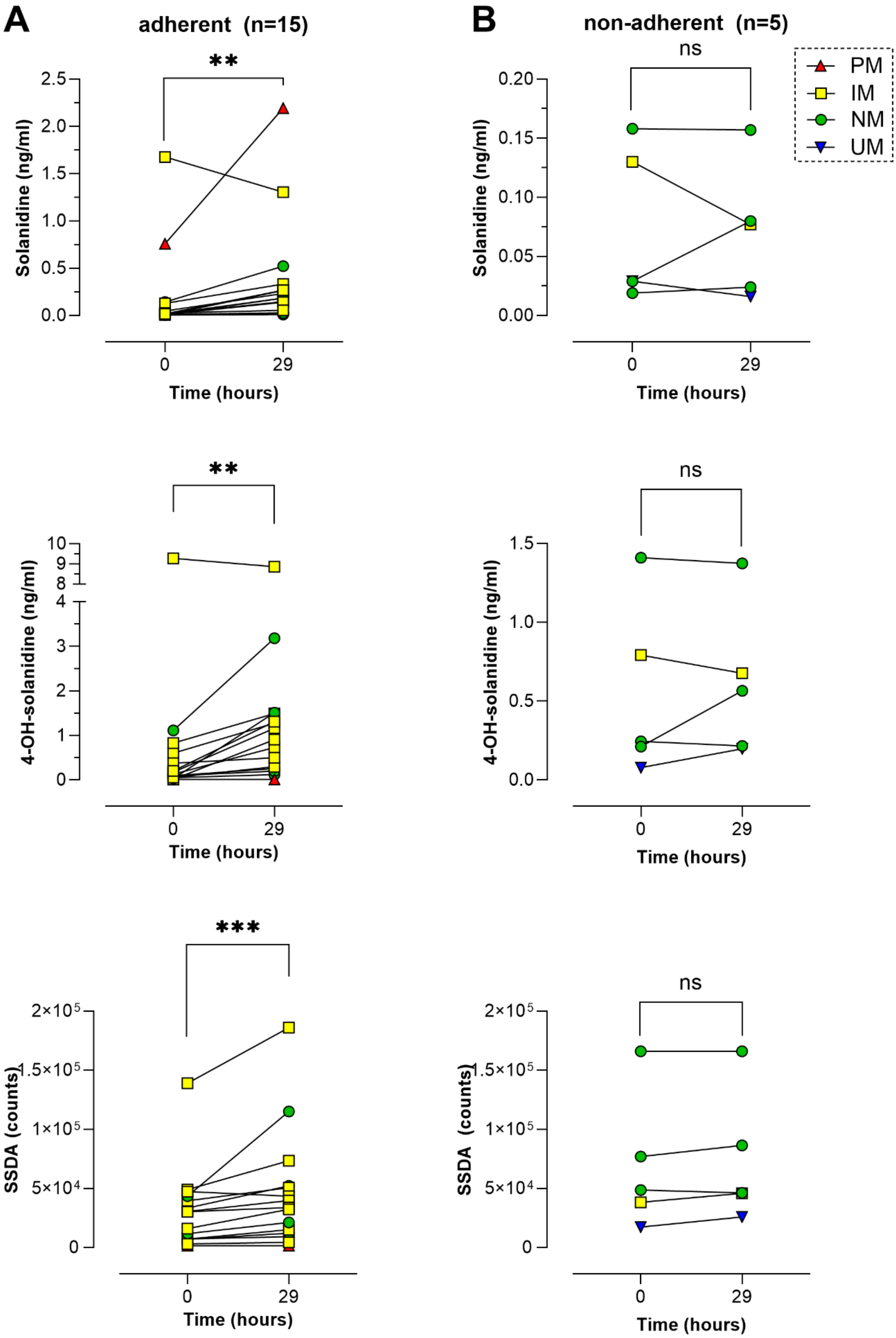

A
adherent (n=15)
B
non-adherent (n=5)
PM
IM
NM
UM
**
**
***
ns
ns
ns
Solanidine (ng/ml)
4-OH-solanidine (ng/ml)
SSDA (counts)
Time (hours)
0
29
2.5
2.0
1.5
1.0
0.5
0.0
0.20
0.15
0.10
0.05
0.00
10
9
8
4
3
2
1
0
1.5
1.0
0.5
0.0
2×10⁵
1.5×10⁵
1×10⁵
5×10⁴
0

Figure S4: Absolute changes in solanidine, 4-OH-solanidine and SSDA plasma concentrations from t0 to t29. A: Participants (n=15) that reported no consumption of potatoes or potato products within three days prior to the study (adherent). B: Participants (n=5) that reported consumption of potatoes or potato products within three days prior to the study (non-adherent). Statistical significance was assessed using Wilcoxon signed-rank test. ns: not significant, **=p-value<0.01, ***=p-value<0.001. Genotype-predicted CYP2D6 phenotypes are depicted by color coding. PM: poor metabolizer, IM: intermediate metabolizer, NM: normal metabolizer, UM: ultrarapid metabolizer

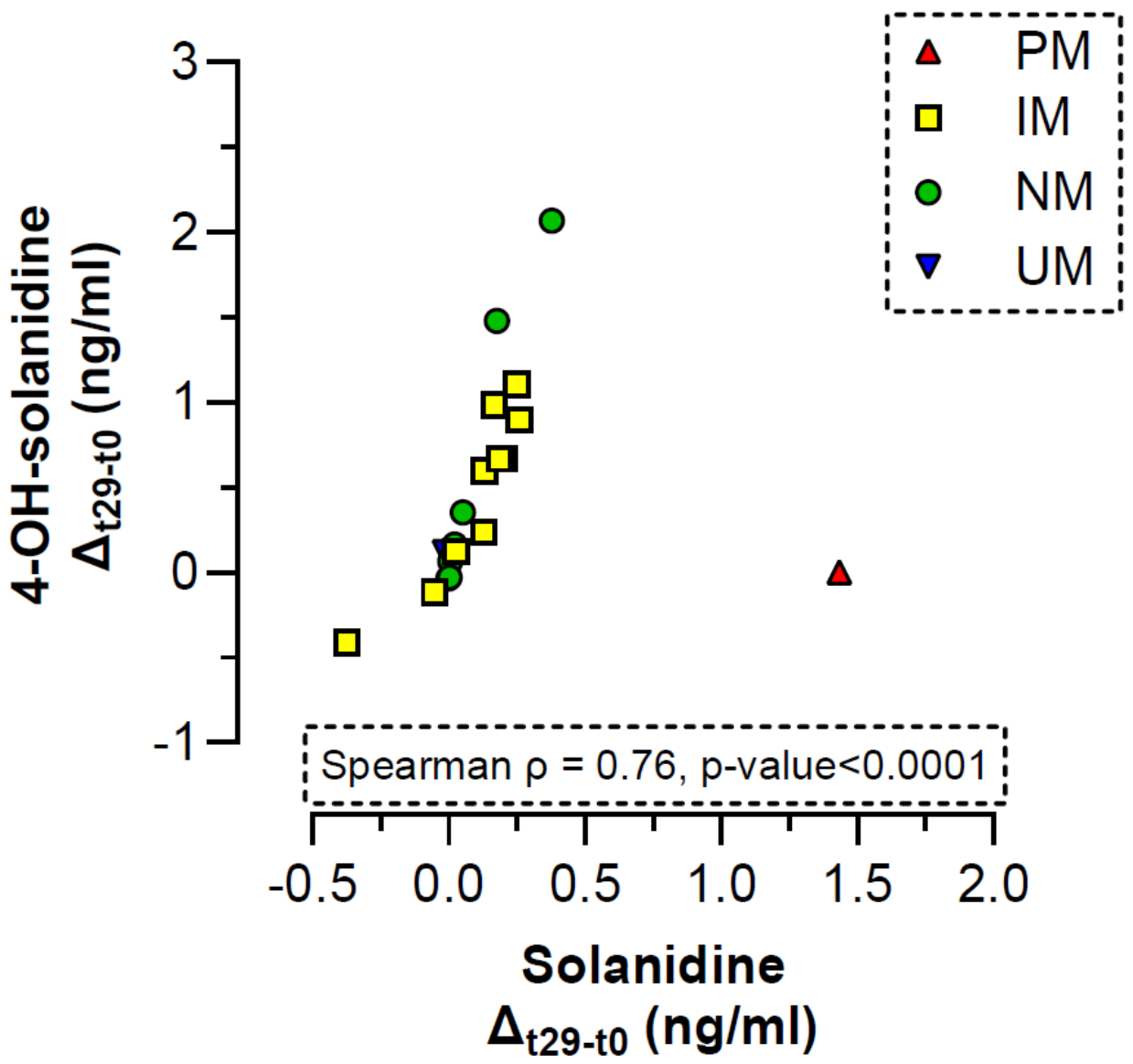

Figure S5: Correlation of solanidine and 4-OH-solanidine absolute changes (t0 to t29). Correlation was assessed using non-parametric Spearman correlation test. Spearman ρ and p-value are given. Genotype-predicted CYP2D6 phenotypes are depicted by color coding. PM: poor metabolizer, IM: intermediate metabolizer, NM: normal metabolizer, UM: ultrarapid metabolizer
